# Alteration of glucocorticoid receptors and exacerbation of inflammation during lytic cytomegalovirus infection in THP‐1 cells

**DOI:** 10.1002/2211-5463.12334

**Published:** 2017-10-30

**Authors:** Shujun Wang, Yaling Dou, Hong Yang, Anping Ni, Rui Zhang, Jiaming Qian

**Affiliations:** ^1^ Department of Gastroenterology Peking Union Medical College Hospital Peking Union Medical College Chinese Academy of Medical Sciences Beijing China; ^2^ Department of Clinical Laboratory Peking Union Medical College Hospital Peking Union Medical College Chinese Academy of Medical Sciences Beijing China

**Keywords:** glucocorticoid receptor, glucocorticoid‐resistant, ulcerative colitis

## Abstract

Cytomegalovirus (CMV) infection is associated with glucocorticoid resistance in ulcerative colitis (UC) and may exacerbate the disease course. However, the underlying pathogenicity remains unclear. The aim of this study was to explore possible underlying mechanisms during CMV latency and lytic infection in the human mononuclear cell line THP‐1. Latent and activated CMV infection cell models were established. We performed real‐time PCR and western blotting to examine changes in glucocorticoid receptors (GRs) during CMV latency and activation. Pro‐inflammatory and anti‐inflammatory cytokines were detected by ELISA. After UV‐inactivated CMV infection, GRs and cytokines were also examined. The expression of GRs was elevated in the reactivation group. An increased ratio of GR β/α and phosphorylation of GRα in the CMV reactivation group may explain refractory response to steroids. During CMV lytic infection, pro‐inflammatory cytokines IL‐6 and TNF‐α increased remarkably and anti‐inflammatory cytokine IL‐5 decreased, which may exacerbate UC. GR and cytokines were unchanged in the UV‐inactivated CMV infection group. Changes in the number and function of GRs may account for glucocorticoid resistance in CMV reactivation. The imbalance of pro‐ and anti‐inflammatory cytokines may be related to severe inflammation.

**Abbreviations**
ATCC: American Type Culture Collection
CMV: cytomegalovirus
CPE: cytopathic effect
DMEM: Dulbecco's modified Eagle's medium
GRs: glucocorticoid receptors
IBD: inflammatory bowel disease
IE: immediate early gene
MOI: multiplicity of infection
pGRα: phosphorylation of GRα
UC: ulcerative colitis

Cytomegalovirus (CMV) is a ubiquitous beta‐herpes virus that infects 70–100% of the general population 1. It often persists lifelong in a latent state after primary infection in humans. However, during conditions of immunosuppression, such as HIV infection or the use of immunosuppressant therapy in inflammatory bowel disease (IBD), CMV can be reactivated and exacerbate the primary disease process. After primary infection, the virus enters a latency phase in endothelial cells, macrophages or granulocyte stem cells. UL82 is expressed in CMV immunoglobulin G antibody‐seropositive donors 2 and is recognized as a latency‐associated gene. Reactivation can be triggered by immune suppressors. During CMV reactivation, the immediate early gene (IE1) initiates viral replication and is a marker for lytic infection. In active ulcerative colitis (UC), monocytes and macrophages infiltrate intestinal lesions 3. THP‐1 cells derived from a patient with acute monocytic leukaemia share similar features with human monocytic cells. THP‐1 can become monocyte‐derived macrophages after PMA stimulation, which can be used to study CMV lytic infections 4. Based on these findings, we established a cell model to mimic CMV latency and lytic infection. However, it is not yet known which mechanisms are involved in the pathogenesis of CMV disease or disease progression.

Glucocorticoids exert potent immunosuppressive effects after binding to glucocorticoid receptors (GRs), ligand‐activated transcription factors. Genetic factors and acquired infections can often affect the efficacy of glucocorticoids. Recent studies showed that CMV reactivation was associated with refractory responses to steroids and may result in disease progression in UC 5, 6, 7. After antiviral therapy, steroid resistance can be reversed 8, and surgery‐free survival can be improved 9, 10, 11. How CMV lytic infection regulates glucocorticoid resistance and disease progression remains unknown. The aim of this study was to reveal possible mechanisms of steroid resistance and inflammation by a refined cell model of CMV latency and reactivation.

## Materials and methods

### Virus, cells and reagents

Cytomegalovirus strain AD169, graciously provided by A.‐p. Ni (Clinical Laboratory Department, Peking Union Medical College Hospital), was obtained from the American Type Culture Collection (ATCC, Manassas, VA, USA). CMV was irradiated with UV light (100 V, 15 W) for 30 min at 8 cm to inactivate CMV AD169. CMV inactivation was confirmed by failure to detect cytopathic effect (CPE) in cells cultured with the inactivated CMV. MRC‐5, human fetal lung fibroblast cells, and THP‐1, a monocytic cell line derived from human acute monocytic leukaemia, were both obtained from ATCC. MRC‐5 cells were cultured in calcium‐free Dulbecco's modified Eagle's medium [DMEM(–Ca)] supplemented with 10% FBS (Gibco, New York, NY, USA), 0.1 mmol per L nonessential amino acids (Invitrogen, Carlsbad, CA, USA). CMV AD169 was propagated in MRC‐5 cells at multiplicity of infection 1 (MOI 1). THP‐1 was grown in RPMI 1640 (HyClone, Logan, UT, USA) supplemented with 10% FBS. PMA, obtained from Sigma‐Aldrich (St. Louis, MO, USA), was used at a final concentration of 100 ng·mL^−1^ for 3 days to induce differentiation of THP‐1‐derived macrophages.

### Infection of THP‐1 cells

Cytomegalovirus infection latency model methods have previously been described 12. In brief, CMV AD169 was added to THP‐1 cells at a MOI of 3. Infected THP‐1 cells were cultured for different times. THP‐1 monocytes were differentiated into macrophage cells by the addition of 100 nm phorbol‐12‐myristate‐13‐acetate, as previously described 13. To model reactivation, we compared the CMV infection latency model with the PMA‐induced differentiation method, PMA differentiation of THP‐1 into macrophages, and then the CMV infection of THP‐1‐derived macrophages method. Each model was detected by observing cell morphology, qRT‐PCR quantification of viral DNA copies in single cells and RT‐PCR detection of viral immediate early (IE) and latency‐related genes.

### RT‐PCR

Total RNA was extracted from cells using Trizol reagent (Invitrogen), and a UV spectrophotometer (NanoDrop ND‐1000, Thermo Fisher Scientific, MA, USA) was used to determine RNA concentration and purity. One microgram of total RNA was reverse‐transcribed to cDNA using MultiScribe Reverse Transcriptase (Applied Biosystems, Foster City, CA, USA) as per the manufacturer's instructions. Reaction product was preserved at −20 °C. Primers were synthesized by Beijing Sangon Biological Engineering Technology Co. (Beijing, China), and the sequences are shown in Table 1. RT‐PCR was performed. Briefly, the target gene was amplified by PCR using the following reaction conditions: initial denaturation at 94 °C for 5 min, denaturation at 94 °C for 30 s, primer annealing at 55 °C for 30 s, elongation at 72 °C for 30 s, all for 30 cycles, final elongation at 72 °C for 10 min. The PCR products were analysed by 1.5% agarose gel electrophoresis, and the results were observed under ultraviolet illumination. When a band clearly appeared in an appropriate size range, it was considered positively amplified. Images of the resulting gels were captured using the UVP gel imaging system (UVP Co. Ltd., Upland, CA, USA). GAPDH was measured to estimate the relative content of the mRNA of each gene. The experiment was performed in triplicate for each sample.

**Table 1:** Primers used in this study

Gene name	Prime sequence (5′–3′)
IE1	
F	ACACGATGGAGTCCTCTGCC
R	TTCTATGCCGCACCATGTCC
UL82	
F	TGCCCTGGATGCGATACTG
R	AGGACCTGACGATGACCCG
GAPDH	
F	CGCTCTCTGCTCCTCCTGTT
R	CCATGGTGTCTGAGCGATGT
GRα	
F	AGCCATTGTCAAGAGGGAAG
R	AGCAATAGTTAAGGAGATTTTCAACC
GRβ	
F	AGCCATTGTCAAGAGGGAAG
R	GCTTTCTGGTTTTAACCACATAAC

### Real‐time quantitative PCR (qPCR)

Based upon the latency and reactivation cell models *in vitro*, THP‐1 cells were divided into four groups: control group, differentiation of THP‐1 with PMA (PMA group), CMV latency infection group (CMV group) and CMV reactivation model (PMA + CMV group). After CMV was inactivated by UV, UV‐inactivated CMV (in‐CMV) replaced CMV in each CMV infection group. After total RNA (1 μg) were converted to cDNA, quantitative PCR (qPCR) was performed with 20 ng of cDNA, 5 μm of primers and 12.5 μL of SYNR green PCR Master Mix (Applied Biosystems) in a total volume of 25 μL. All qPCRs were conducted in triplicate. Sequences of the primers used in qPCR are listed in Table 1. Ct values of the examined molecules were normalized with those of GAPDH, and fold changes were obtained by using the comparative Ct method.

### Measurement of cytokine levels

Supernatants of each group were collected at indicated time points and stored at −70 °C. Samples thawed on ice were subjected to analysis using Biolegend LEGEND plex™ Human Th1/Th2 Panel (8‐plex; BioLegend, San Diego, CA, USA). The assays were performed as per the manufacturers' instructions.

### Statistical analysis

Means and corresponding standard errors in the CMV infection group were compared with uninfected cell values. The two‐sample *t*‐test or one‐way ANOVA test was used to test for differences between two or more groups. A chi‐square or Fisher's exact test was used for the comparison of categorical variables. A *P* value < 0.05 was the criterion for a statistically significant difference.

## Results

### Establishment of latent and lytic CMV infection in THP‐1

**Figure 1:**
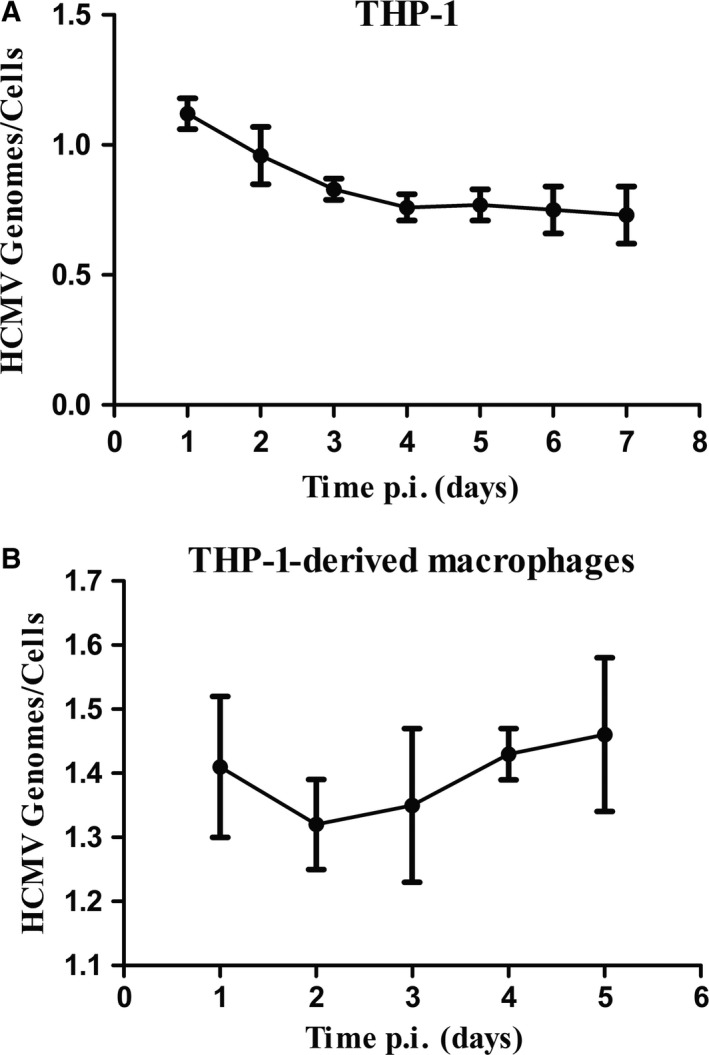
Intracellular CMV genome copies. (A) CMV genome copies in THP‐1 cell line. (B) THP‐1 cells were differentiated to THP‐1‐derived macrophages after stimulation with 100 nm PMA. CMV genome copies were also determined.

**Figure 2:**
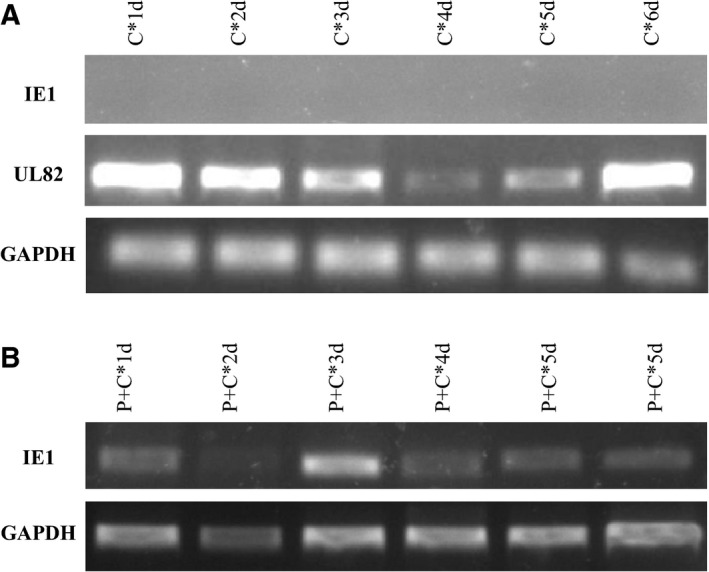
mRNA expression of IE1 with or without latency‐associated gene UL82 at various time points after infection. (A) UL82 expressed in THP‐1, while IE1 was not detected. (B) The expression of IE1 in THP‐1‐derived macrophages, with CMV infection for 3 days, was significant.

THP‐1 monocyte cell line was grown in suspension after CMV infection. Three days later, qRT‐PCR quantifying CMV viral DNA copies became stable (Fig. 1A) in THP‐1. The latency‐associated gene UL82 expressed after CMV infection, while the IE1 gene was not detected (Fig. 2A). Stable CMV DNA copies and the expression of latency‐associated genes demonstrated that CMV was in latency.

Virus load increased after infection for 2 days (Fig. 1B), and IE1 expressed after CMV infected THP‐1‐derived macrophages (Fig. 2B). The expression of IE1 was remarkable 3 days after infection. These observations suggest a reactivation model was successfully established. Thus, we selected CMV infection for 3 days as the time point for both latency (CMV) and lytic infection (P + C below). After CMV was inactivated by UV, UV‐inactivated CMV (in‐CMV) replaced CMV in each CMV infection group.

### Altered expression of glucocorticoid receptors during CMV latent and lytic infection

**Figure 3:**
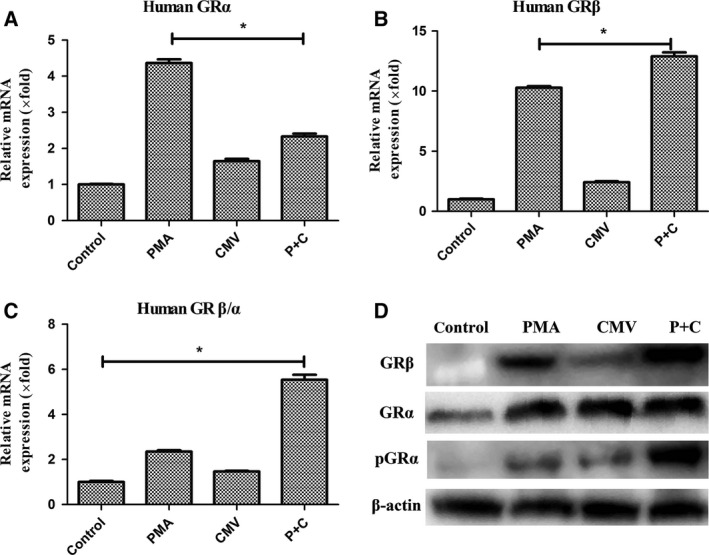
mRNA expression of GR during CMV latency and lytic infection. (A) Relative mRNA expression of GRα in each group. (B) Relative mRNA expression of GRβ. (C) mRNA ratio of GRβ to GRα. Bars represent means and standard errors of fold changes in mRNA expression in each group from three independent experiments. *P < 0.05. (D) Western blots of GR and Ser226 phosphorylated protein of GRα.

In this study, compared with the control group, relative mRNA expression of human GRα and GRβ was elevated in each group. GRα expression in the PMA group increased more significantly than that in the CMV lytic infection group, while GRβ expression was most significant in the CMV lytic group (Fig. 3A,B). The GRβ/α ratio increased significantly in the CMV lytic infection group compared with that in the control group (5.54 ± 0.22, *P *=* *0.035; Fig. 3C). The significant increase in GRβ/α during reactivation may explain refractory response to steroid treatment.

Glucocorticoid receptor α can be regulated by complex cellular mechanisms. Phosphorylation of GRα at serine 226 (pGRα‐Ser226) could contribute to glucocorticoid resistance by enhancing nuclear export of GR and inhibiting GR transcriptional activity 14. However, pGRα‐Ser226 could increase the mRNA level of GRα. We also detected pGRα‐Ser226 by western blotting. pGRα increased in the lytic infection group (Fig. 3D). This may indicate that during lytic infection, increased pGRα may alter glucocorticoid response.

### CMV lytic infection exacerbates inflammation

**Figure 4:**
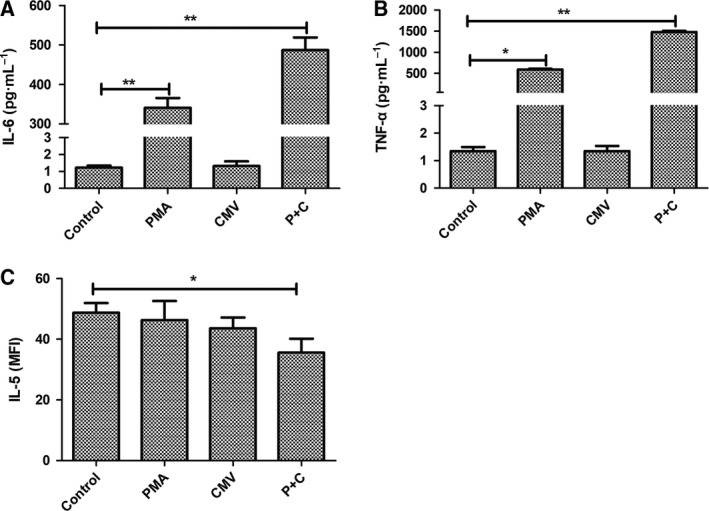
Cytokine concentrations were measured from the culture supernatants. The columns show the averages, and the error bars indicate the standard deviation of three replicate samples in each group. *P < 0.05. **P < 0.01 compared two groups indicated.

To explore possible mechanisms of disease exacerbation during CMV lytic infection, we evaluated several pro‐ and anti‐inflammatory cytokines from culture supernatants. Our results showed that the PMA group and the CMV lytic infection group had increased pro‐inflammatory cytokines IL‐6 (PMA: 340.76 ± 24.60 vs control: 1.22 ± 0.02 pg·mL^−1^, *P *=* *0.006; PMA + CMV: 487 ± 31.50 pg·mL^−1^, *P *=* *0.004; Fig. 4A) and TNF‐α (PMA: 589.29 ± 21.25 pg·mL^−1^ vs control: 1.35 ± 0.15 pg·mL^−1^, *P *=* *0.003; PMA + CMV: 1478 ± 29.80, *P *=* *0.002; Fig. 4B). Furthermore, the expression of pro‐inflammatory cytokines in the CMV lytic infection group was greater than that measured in the PMA group. CMV infection latency had no significant effect (IL‐6: 1.32 ± 0.27, *P *=* *0.734; TNF‐α: 1.35 ± 0.19, *P *=* *0.632). Anti‐inflammatory cytokine IL‐5 decreased in the CMV lytic infection group (35.6 ± 4.6 vs control 48.7 ± 3.2, *P *=* *0.032; Fig. 4C). During lytic infection, increased pro‐inflammatory cytokines IL‐6 and TNF‐α and decreased anti‐inflammatory cytokine IL‐5 may exacerbate UC.

### The reversal effects of GR and cytokines after CMV inactivation

**Figure 5:**
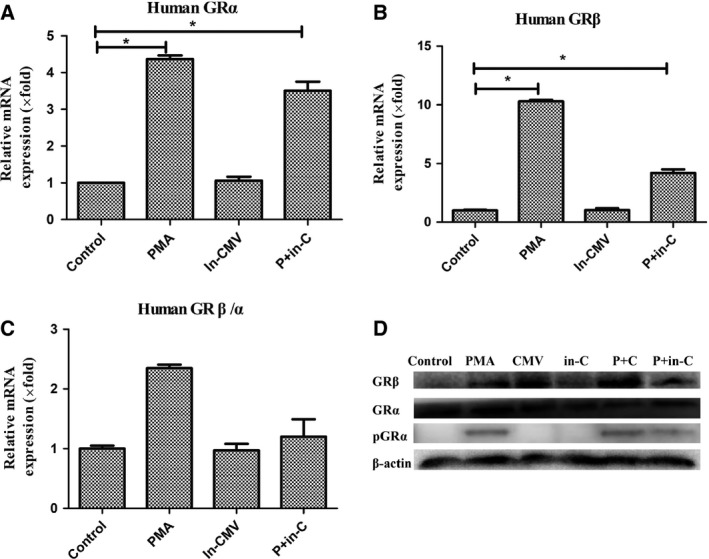
mRNA expression of GR in CMV mock latency and lytic infection. (A) Relative mRNA expression of GRα in each group. (B) Relative mRNA expression of GRβ. (C) mRNA ratio of GRβ to α. Bars represent means and standard errors of fold changes in mRNA expression in each group in three independent experiments. *P < 0.05. (D) Western blots of GR and Ser226 phosphorylated protein of GRα.

**Figure 6:**
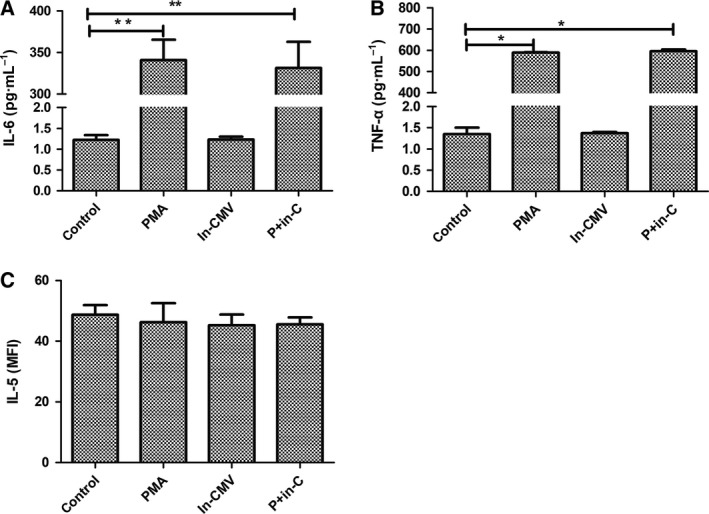
Cytokine concentrations were measured from the culture supernatants. The columns show the averages, and the error bars indicate the standard deviation of three replicate samples in each group. *P < 0.05. **P < 0.01 compared two groups indicated.

To examine potential reversal impact of CMV infection on steroid resistance and cytokines, we identified GRs and cytokines differentially expressed in mock‐infected latency (inactivated CMV) and mock‐infected lytic infection groups (P + in‐C). There was no significant change in GRs (Fig. 5A–D) or cytokines (Fig. 6A–C) during mock‐infected latency. No significant differences in the mock lytic infection and PMA groups were observed.

## Discussion

We demonstrate during CMV lytic infection an increased ratio of GR β to α, together with increased phosphorylation of GR α, leading to glucocorticoid resistance. Increased pro‐inflammatory and decreased anti‐inflammatory cytokines may progress disease course. However, in CMV latency and UV‐inactivated CMV infection, neither significant change in GRs nor imbalance of cytokines can be observed. The processes observed by our study are compatible with current clinical consensus, providing a strategy to treat UC patients with CMV lytic infection. Antiviral therapy to inactivate CMV lytic infection in UC patients with glucocorticoid resistance may offer a potential advantage in clinical applications. After antiviral therapy, steroid resistance can be reversed, and glucocorticoid can then be used to treat activated disease, avoiding high doses of glucocorticoid and invasive surgery. ECOCC consensus has established that only after antiviral therapy in UC patients with CMV lytic infection can immune suppressants be administered 15.

We describe a CMV latency and lytic infection cell model *in vitro* that applies CMV infection to a wide scope of inflammatory diseases. The role of CMV infection in clinical UC patients has been fully elucidated compared with *in vitro* research. A lack of a proper *in vitro* cell models has posed challenges. Thus, we first established a CMV infection cell model and then applied UV‐inactivated CMV infection. Our latency model is in accordance with a previous study 12. However, CMV lytic infection model used different methods. So far, there was no study to compare different method. Besides, studies on CMV lytic infection model often use different CMV strains such as Towne strain 16 or pretreated with glucocorticoids 17, 18. Use of steroids may disturb expression of GRs. So we investigate a new CMV lytic infection model without the use of glucocorticoid. After PMA induction of THP‐1 differentiation, CMV infection could become a lytic infection. In our study, both CMV DNA copies and lytic associated gene IE1 were detected to build a more precise lytic infection model. This model applies universally to CMV infection‐associated diseases.

Clinical use of glucocorticoid is hampered by steroid resistance. In UC patients, CMV infection is associated with glucocorticoid resistance via an unclear mechanism. Glucocorticoids enter cells, bind to GR α and translocate into the nucleus. Steroids can then increase anti‐inflammatory cytokines and decrease pro‐inflammatory cytokines. Two primary GRs are GRα and β. GCs bound to GRα can subsequently exert anti‐inflammatory effects. GCs bound to GRβ cannot exert anti‐inflammatory effects. Increased GRβ is associated with glucocorticoid resistance 19, 20. In our study, we found that CMV lytic infection could increase the ratio of GR β to α, which is associated with corticoid resistance in UC patients 19. It is reported that phosphorylation of serine 226 in GR α can result in glucocorticoid resistance 14. As far as we know, our study is the first to demonstrate that serine 226 pGRα together with increased GRβ is associated with glucocorticoid resistance in UC patients with CMV lytic infection.

The impact of CMV infection, especially lytic infection, in UC patients is still unclear. Several studies indicate that CMV infection can exacerbate colonic inflammation and the need for surgery 6, 11. However, some studies insist CMV is merely a ‘bystander’ during UC 21. Alterations in pro‐ and anti‐inflammatory cytokines secreted by monocytes and macrophages play a role in the course of UC 3, 22. Furthermore, CMV infection can also induce CMV‐related colitis. To investigate the role of CMV in UC, we measured cytokines after CMV infection. In our study, pro‐inflammatory cytokines IL‐6 and TNF‐α increased during CMV lytic infection. During infection latency, cytokines were not significantly changed. TNF‐α was reported to increase CMV lytic infection 23. This may become a vicious cycle. Anti‐inflammatory cytokine IL‐5 decreased after CMV lytic infection. Changes in cytokines may indicate the mechanism of how CMV lytic infection worsens UC. UC exacerbation after CMV infection may also account for refractory responses to steroid treatment.

In conclusion, we established CMV latency and lytic infection models *in vitro* and preliminarily explored a possible mechanism of CMV infection in glucocorticoid resistance and cytokine production. These findings provide insight and suggest an association between CMV infection and steroid‐resistant UC. However, how CMV infection results in changes in GRs and cytokines and whether cytokines alterations are associated with changes in GRs were not considered in this study, and further investigation is needed.

## Author contributions

JQ conceived of and designed the experiments. SW, YD, AN and RZ performed the experiments. SW and HY analysed the data. YD, AN and RZ contributed reagents/materials. SW and HY wrote the manuscript.
